# Effect of a potassium-competitive acid blocker on the gut microbiota in patients undergoing percutaneous coronary intervention: a randomized pilot study

**DOI:** 10.3389/fcimb.2026.1942212

**Published:** 2026-10-12

**Authors:** Sang Hoon Lee, Seong-In Na, Sung Chul Park, Byung Ryul Cho, Bong Ki Lee, Yong-Joon Cho, Hyunseok Cho, Hee-Jeong Youk, Dong Ryeol Ryu

**Affiliations:** 1 Department of Internal Medicine, Kangwon National University Hospital, Kangwon National University School of Medicine, Chuncheon, Republic of Korea; 2 Multidimensional Genomics Research Center, Kangwon National University, Chuncheon, Republic of Korea; 3 Department of Molecular Bioscience, College of Biomedical Science, Kangwon National University, Chuncheon, Republic of Korea; 4 Department of Emergency Medicine, Kangwon National University Hospital, Kangwon National University School of Medicine, Chuncheon, Republic of Korea; 5 Department of Laboratory Medicine, Kangwon National University Hospital, Kangwon National University School of Medicine, Chuncheon, Republic of Korea

**Keywords:** gut microbiota, Lachnospiraceae, percutaneous coronary intervention, potassium-competitive acid blocker, proton pump inhibitor, *Streptococcus*

## Abstract

**Background and aim:**

Proton pump inhibitors (PPIs) are routinely used in patients undergoing percutaneous coronary intervention (PCI) to prevent gastrointestinal bleeding associated with dual-antiplatelet therapy (DAPT). However, long-term PPI use has been linked to gut microbiota dysbiosis. Tegoprazan, a potassium-competitive acid blocker (P-CAB), may exert distinct effects on the gut microbiome. This study compared the longitudinal effects of tegoprazan and pantoprazole on gut microbiota composition in patients with angina undergoing PCI.

**Methods:**

This prospective, randomized, controlled pilot study randomized 38 patients 1:1:1 to tegoprazan 50 mg once daily, pantoprazole 40 mg once daily, or no acid-suppressive therapy for 24 weeks. Five participants did not provide baseline fecal samples, and nine discontinued participation before week 24. Thus, 24 participants with paired baseline and week-24 fecal samples were included in the final analysis. Fecal samples were analyzed using 16S rRNA gene sequencing.

**Results:**

No drug-related adverse events or gastrointestinal bleeding occurred in any group. Observed amplicon sequence variant (ASV) richness was significantly higher at week 24 than at baseline in the overall cohort (p = 0.003) and P-CAB group (p = 0.016), whereas the Shannon index showed no significant changes. Paired PERMANOVA with permutations restricted by patient ID revealed significant differences in Bray–Curtis beta diversity between baseline and week 24 in the P-CAB group (R² = 0.070, p = 0.031) and PPI group (R² = 0.059, p = 0.008), but not in the no acid-suppressive therapy group (R² = 0.021, p = 0.691). In the formal cross-arm analysis, the treatment group-by-visit interaction was not significant for Bray–Curtis beta diversity (R² = 0.021, pseudo-F = 0.505, p = 0.270). Although nominal within-arm taxonomic changes were observed, no treatment group-by-visit interaction survived Benjamini–Hochberg correction in ANCOM-BC2.

**Conclusion:**

Tegoprazan was well tolerated, with no drug-related adverse events or gastrointestinal bleeding during the 24-week follow-up. Both acid-suppressive treatment groups showed modest longitudinal microbiome changes. The taxon-level findings were exploratory and hypothesis-generating and should not be interpreted as definitive evidence of treatment-specific differential effects. Larger, adequately powered studies are required to confirm these observations.

## 1 Introduction

Dual antiplatelet therapy (DAPT) is commonly used in patients undergoing percutaneous coronary intervention (PCI) to prevent stent thrombosis and recurrent cardiovascular events. DAPT is typically continued for a defined period, individualized according to the patient’s ischemic and bleeding risks (Knuuti et al., 2020; Nakamura et al., 2020). Considering the increased risk of gastrointestinal (GI) bleeding associated with long-term antiplatelet therapy, concomitant acid suppression therapy for gastroprotection is considered particularly in patients at a high risk of GI bleeding (Abraham et al., 2010; Scally et al., 2018). Proton pump inhibitors (PPIs) are commonly considered for GI protection in high-risk patients receiving antiplatelet therapy (Bhatt et al., 2008), as they have been shown to significantly reduce the incidence of gastrointestinal events, regardless of the intensity of antiplatelet regimens (Vaduganathan et al., 2016).

Although pantoprazole and rabeprazole have been suggested to have a lower potential for CYP2C19-mediated interactions with clopidogrel than omeprazole, the clinical significance of such interactions remains controversial (Melloni et al., 2015; Vaduganathan et al., 2016). Although PPIs effectively reduce the risk of GI bleeding, their long-term use has been associated with potential adverse effects (Moayyedi et al., 2019), including bone fractures, *Clostridioides difficile* infection, community-acquired pneumonia, and alterations in the gut microbiota composition (Eom et al., 2011; Kwok et al., 2011; Deshpande et al., 2012; Imhann et al., 2016). Therefore, PPI prescriptions should be individualized by balancing the benefits of GI protection against the potential risks for each patient.

Tegoprazan is a novel potassium-competitive acid blocker (P-CAB) that reversibly inhibits gastric H^+^/K^+^-ATPase (Takahashi and Take, 2018). Unlike PPIs, P-CABs do not require acid activation and are metabolized predominantly by CYP3A4 rather than CYP2C19, thereby theoretically reducing their interactions with CYP2C19-dependent antiplatelet agents such as clopidogrel (Yang et al., 2023; Scarpignato and Hunt, 2024). Preclinical studies have suggested that tegoprazan may preserve gut microbiota diversity and maintain intestinal barrier function better than rabeprazole; however, further clinical validation is needed. Given the differences in their mechanisms of action and metabolic pathways, tegoprazan may have distinct effects on the gut microbiota compared to conventional PPIs (Son et al., 2022). However, data on the effect of tegoprazan on the gut microbiota in clinical populations, particularly in patients undergoing PCIs requiring long-term acid suppression therapy, remain limited.

Beyond alterations in taxonomic composition, the gut microbiota may influence host physiology through microbial metabolites and microbe–host signaling pathways (Andalib et al., 2025). Short-chain fatty acids generated by bacterial fermentation contribute to epithelial barrier homeostasis and immunometabolic regulation (Koh et al., 2016), whereas other microbiota-derived metabolites, including trimethylamine N-oxide (TMAO) and secondary bile acid derivatives, have been associated with vascular inflammation, endothelial dysfunction, platelet activation, thrombosis and cardiovascular physiology (Witkowski et al., 2020; Ronen et al., 2024). These functional interactions may be particularly relevant in patients undergoing PCI, in whom inflammatory and thrombotic pathways contribute to subsequent cardiovascular risk. Accordingly, acid suppression-associated alterations in the gut microbiota may have implications extending beyond gastrointestinal safety to the broader gut–heart axis.

In this prospective, randomized pilot study, we compared the gut microbiota composition by performing 16S rRNA gene sequencing in three groups: P-CAB, PPI, and no acid-suppressive therapy groups.

## 2 Materials and methods

### 2.1 Study design and participants

This was a prospective, randomized, open-label, single-center pilot study conducted at Kangwon National University Hospital (Chuncheon, South Korea). Eligible participants were patients aged ≥ 18 years diagnosed with angina pectoris who underwent PCI with stent placement and who required DAPT for ≥6 months post-procedure. Participants were required to be enrolled within 1 week of PCI. Exclusion criteria included: (1) prior use of PPIs, P-CABs, or H2-receptor antagonists within 4 weeks; (2) antibiotic or probiotic/prebiotic use within 4 weeks; (3) active malignancy or history of malignancy within 5 years (with exceptions for complete remission [CR] and endoscopic submucosal dissection [ESD]-treated cases); (4) major adverse events within 3 months (myocardial infarction [MI], high-degree atrioventricular [AV] block, acute delirium, paralytic ileus); (5) hepatic or renal dysfunction (aspartate aminotransferase [AST], alanine aminotransferase [ALT], alkaline phosphatase [ALP], total bilirubin, blood urea nitrogen [BUN], or creatinine levels > 2 × upper limit of normal); (6) significant psychiatric illness precluding stool sample collection; (7) chronic nonsteroidal anti-inflammatory drug (NSAID), corticosteroid, or anticoagulant use; and (8) pregnancy or lactation. All participants provided written informed consent after receiving a detailed explanation of the study protocol. As this was an exploratory pilot study, no formal power-based sample-size calculation was performed. At the time of study planning, sufficient prior clinical data were unavailable to reliably estimate an expected effect size for longitudinal changes in the gut microbiota among the three treatment groups. The target sample size was set at 39 participants (13 per group), determined pragmatically based on study feasibility, anticipated recruitment, and the resources required for microbiome analyses. A total of 38 participants were ultimately assessed for eligibility and randomized within the recruitment period (**Figure 1A**).

**Figure 1:**
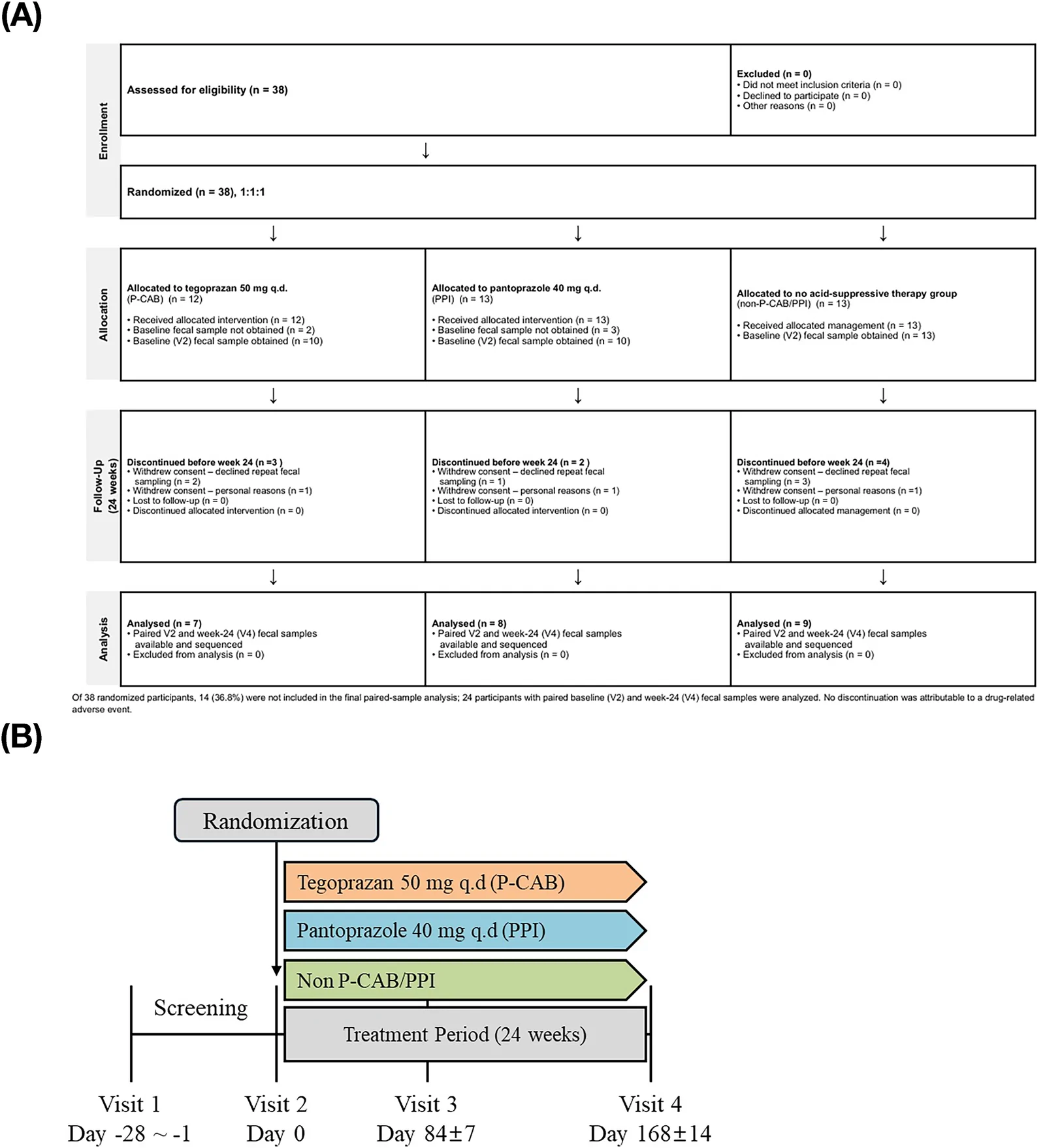
Study design and participant flow. **(A)** CONSORT flow diagram of participant enrollment, allocation, follow-up, and analysis. A total of 38 participants were assessed for eligibility and randomized to the P-CAB group (tegoprazan 50 mg once daily; n = 12), PPI group (pantoprazole 40 mg once daily; n = 13), or the no acid-suppressive therapy group (n = 13). Baseline fecal samples were not obtained from two participants in the P-CAB group and three in the PPI group. Among the remaining 33 participants, nine participants discontinued participation before week 24. In the P-CAB group, two participants declined repeat fecal sampling and one withdrew for personal reasons; in the PPI group, one declined repeat fecal sampling and one withdrew for personal reasons; and in the no acid-suppressive therapy group, three declined repeat fecal sampling and one withdrew for personal reasons. Consequently, 24 participants with paired baseline (V2) and week-24 (V4) fecal samples were included in the final analysis: seven in the P-CAB group, eight in the PPI group, and nine in the no acid-suppressive therapy group. No discontinuation was attributable to a drug-related adverse event. **(B)** Schematic overview of the study protocol, including randomization to the P-CAB, PPI, and no acid-suppressive therapy groups, treatment duration, and fecal sample collection at baseline (V2) and week 24 (V4). P-CAB, potassium-competitive acid blocker; PPI, proton pump inhibitor.

### 2.2 Randomization and study protocol

Patients meeting the eligibility criteria were randomized in a 1:1:1 ratio to the three groups: (1) P-CAB group (tegoprazan 50 mg once daily), (2) PPI group (pantoprazole 40 mg once daily), and (3) no acid-suppressive therapy group. Block randomization with a fixed block size of six was used, with two participants assigned to each study group within each block. The random allocation sequence was generated using SPSS by a study investigator (H.C.) who was not involved in participant enrollment. After eligibility was confirmed, participants were assigned sequential registration numbers, and the allocation schedule was maintained and implemented by a research nurse. The principal investigator and investigators involved in participant enrollment did not have access to upcoming treatment assignments before allocation, thereby maintaining allocation concealment until assignment. Because this was an open-label study, treatment allocation was known to the investigators and participants after assignment. The study medications were administered once daily before meals for 24 weeks (**Figure 1B**). Written informed consent was obtained from all participants. This study was approved by the Institutional Review Board of Kangwon National University Hospital (KNUH-A-2021-12-014-016). The study was registered with the Clinical Research Information Service of the Republic of Korea (KCT0008181).

### 2.3 Data collection and baseline assessments

All participants underwent baseline assessments including medical history, concomitant medications, metabolic indices, and anthropometric measurements. Body mass index (BMI) was calculated as weight (kg) divided by height (m^2^). Metabolic parameters such as blood pressure, heart rate, white blood cell count (WBC), hemoglobin (Hb), platelet count, BUN, creatinine, total bilirubin, AST, ALT, ALP, total protein, albumin, hemoglobin A1c, fasting blood glucose, C-reactive protein (CRP), magnesium (Mg) and lipid profile levels (total, low-density lipoprotein, high-density lipoprotein cholesterol, and triglycerides) were measured. Throughout the 24-week follow-up period, interval medication exposure was monitored using the institutional electronic medical record and prescription-history system. Medication histories were reviewed through the end of follow-up, with particular attention to antibiotics, probiotics/prebiotics, H2-receptor antagonists, additional acid-suppressive medications outside the assigned study treatment, and other medications prohibited by the study protocol. None of the 24 participants included in the final analysis received antibiotics, probiotics/prebiotics, H2-receptor antagonists, additional acid-suppressive medications, or other prespecified prohibited medications during follow-up.

### 2.4 Stool sample collection

Stool samples from all patients who agreed to participate in the study were collected at baseline (V2, day 0, prior to treatment initiation) and at week 24 (V4, 168 ± 14 days). Stool samples were immediately stored on ice and subsequently frozen at −80 °C until DNA extraction and microbiome analysis.

### 2.5 DNA extraction and 16S rRNA gene sequencing

The collected stool samples were subjected to 16S rRNA gene sequencing at Macrogen (Seoul, South Korea) and confirmed by the Multidimensional Genomics Research Center, Kangwon National University. Genomic DNA was extracted from fecal samples using the Qiagen QIAamp DNA Stool Mini Kit (Qiagen, Hilden, Germany), according to the manufacturer’s instructions. DNA concentration was quantified using VICTOR Nivo™ (PerkinElmer, Waltham, MA, USA) with the PicoGreen dsDNA quantification assay.

Sequencing libraries were prepared following the standard Illumina 16S Metagenomic Sequencing Library Preparation protocol targeting the V3–V4 region of the bacterial 16S rRNA gene. Briefly, 5 ng of input genomic DNA was subjected to PCR amplification using universal primers containing Illumina adapter overhang sequences. Amplicons were purified using AMPure XP beads (Beckman Coulter, Brea, CA, USA), followed by a second round of PCR for index incorporation using Nextera XT indexed primers.

The final libraries were purified and quantified using the PicoGreen assay, and quality was checked using the TapeStation D1000 ScreenTape system (Agilent Technologies, Waldbronn, Germany). Libraries were normalized, pooled, and quantified by qPCR using a KAPA Library Quantification Kit (Roche, Basel, Switzerland). Paired-end sequencing was performed using the MiSeq platform (Illumina, San Diego, CA, USA).

### 2.6 Bioinformatic processing and microbial community analysis

Demultiplexed paired-end reads targeting the V3–V4 region of the bacterial 16S rRNA gene were primer- and quality-trimmed using Cutadapt v4.6. Read pairs lacking the expected primer sequences were discarded. Trimmed read pairs were merged using PEAR v0.9.6 with a minimum assembled-read length of 200 bp. The minimum-overlap parameter was not explicitly specified and therefore remained at the PEAR default of 10 bp.

Merged reads were imported into QIIME2 v2024.2 and initially filtered using quality-filter q-score (min-quality = 4, quality-window = 3, min-length-fraction = 0.75, max-ambiguous = 0). ASVs were inferred using q2-dada2 v2024.2 (DADA2 v1.26.0) in single-end mode with trim-left = 0, trunc-len = 0, maxEE = 2, truncQ = 2, and pseudo-pooling; chimeras were removed using the consensus method.

Across 48 samples, 4,434,099 raw read pairs were generated, yielding 2,164,903 non-chimeric reads and 13,849 ASVs after processing. The median final sequencing depth was 45,367 reads per sample (range, 25,562–65,152), with no samples excluded because of insufficient sequencing depth. Detailed read-processing statistics are provided in **Supplementary Table S5**.

Taxonomic assignment of representative ASV sequences was performed using a Naive Bayes classifier trained on the SILVA 138 reference database within the QIIME2 framework. The resulting ASV abundance table, taxonomic assignment table, and sample metadata were imported into SimpleMicrobiome (Na et al., 2026), an R Shiny-based platform, for downstream microbiome analysis and visualization. Data handling within SimpleMicrobiome was based on the phyloseq framework (McMurdie and Holmes, 2013). Taxonomic profiles were summarized at the family and genus levels and converted to relative abundances for visualization. For genus-level visualization and differential-abundance reporting, features without an assigned genus-level annotation were labeled using the closest available higher taxonomic rank. Taxa with a mean relative abundance below 1% across samples were grouped into the “Others” category.

Alpha diversity was evaluated using the Observed ASVs and the Shannon diversity index after rarefaction to 25,562 reads per sample, corresponding to the minimum sequencing depth among the 48 samples. Rarefaction was performed without replacement using a random seed of 42. Beta diversity was assessed based on Bray–Curtis dissimilarity and visualized using principal coordinate analysis (PCoA). Statistical analyses and visualizations were performed using vegan and ggplot2 (Wickham, 2016).

### 2.7 Statistical analyses

The prespecified primary efficacy endpoint was broadly defined as the difference in the overall gut microbiota profile among the three treatment groups after 24 weeks of treatment, based on 16S rRNA sequencing. The secondary efficacy endpoint was the occurrence of gastrointestinal bleeding. The original protocol did not designate a single quantitative microbiome metric as the primary endpoint. Accordingly, given the exploratory pilot design and small sample size, the detailed microbiome analyses performed in the present study—including α-diversity, β-diversity, and genus-level differential-abundance analyses—were considered exploratory and hypothesis-generating. Multiple-testing correction using the Benjamini–Hochberg method was applied where appropriate, particularly for taxon-level analyses. For baseline comparisons among the three treatment groups (**Table 1**), continuous variables are presented as median (Q1, Q3) and were compared using the Kruskal–Wallis test. Fisher’s exact test was used to compare categorical variables among the three groups. Statistical analyses were performed using SPSS for Windows (version 19.0; IBM Corp., Armonk, NY, USA). Statistical significance was set at *p* < 0.05.

**Table 1:** Demographic and clinical characteristics of the study population (N = 24).

Characteristic	P-CAB	PPI	No acid-suppressive therapy group	Total	*p*-value
	(n=7)	(n=8)	(n=9)	(n=24)	
Male sex, n (%)	6 (85.7%)	5 (62.5%)	7 (77.8%)	18 (75.0%)	0.670
Age (years)	72.0 (68.5, 78.0)	59.5 (59.0, 62.2)	70.0 (66.0, 76.0)	68.5 (62.8, 74.5)	0.004
Height (cm)	165.0 (158.1, 166.2)	171.6 (160.1, 176.2)	157.5 (151.9, 162.0)	161.5 (156.1, 169.5)	0.065
Weight (kg)	65.6 (57.2, 73.2)	65.5 (60.3, 69.5)	62.7 (61.0, 67.0)	64.7 (59.4, 70.0)	0.902
Alcohol consumption, n (%)	2 (28.6%)	5 (62.5%)	3 (33.3%)	10 (41.7%)	0.336
Smoking status, n (%)	1 (14.3%)	4 (50.0%)	1 (11.1%)	6 (25.0%)	0.134
Vital signs					
SBP (mmHg)	137 (132, 142)	129 (120, 134)	132 (125, 133)	132 (125, 137)	0.124
DBP (mmHg)	85 (80, 88)	80 (72, 86)	76 (74, 88)	80 (74, 88)	0.458
HR (beats/min)	68 (66, 70)	78 (70, 81)	71 (63, 77)	70 (67, 78)	0.248
RR (breaths/min)	20 (20, 20)	20 (20, 20)	20 (20, 20)	20 (20, 20)	
BT (°C)	36.5 (36.5, 36.6)	36.5 (36.5, 36.8)	36.6 (36.5, 36.6)	36.5 (36.5, 36.6)	0.955
Underlying disease					
HTN	5 (71.4%)	3 (37.5%)	4 (44.4%)	12 (50.0%)	0.387
DM	2 (28.6%)	1 (12.5%)	3 (33.3%)	6 (25.0%)	0.592
Dyslipidemia	4 (57.1%)	1 (12.5%)	2 (22.2%)	7 (29.2%)	0.14
CVD	0 (0.0%)	0 (0.0%)	1 (11.1%)	1 (4.2%)	0.419
CLD	0 (0.0%)	0 (0.0%)	0 (0.0%)	0 (0.0%)	_
CKD	0 (0.0%)	0 (0.0%)	0 (0.0%)	0 (0.0%)	_
CRD	0 (0.0%)	0 (0.0%)	2 (22.2%)	2 (8.3%)	0.162
Laboratory findings					
WBC (×10^3^/μL)	8.40 (6.05, 8.75)	6.45 (5.38, 7.68)	6.30 (5.00, 7.00)	6.40 (5.45, 8.43)	0.729
Hemoglobin (g/dL)	14.2 (13.9, 15.2)	15.1 (13.7, 15.3)	13.7 (13.1, 14.6)	14.3 (13.6, 15.3)	0.627
Platelet (×10^3^/μL)	202 (181, 236)	198 (166, 238)	179 (167, 186)	185 (175, 214)	0.247
Blood Urea Nitrogen (mg/dL)	15.8 (14.8, 20.4)	12.0 (10.4, 13.6)	14.1 (13.2, 17.4)	14.1 (12.9, 17.0)	0.012
Creatinine (mg/dL)	0.87 (0.78, 0.89)	0.77 (0.72, 0.83)	0.84 (0.79, 1.06)	0.82 (0.75, 0.92)	0.497
Total Bilirubin (mg/dL)	0.65 (0.55, 0.68)	0.65 (0.47, 0.79)	0.66 (0.44, 0.96)	0.65 (0.47, 0.78)	0.996
AST (U/L)	31 (22, 34)	28 (26, 32)	24 (22, 41)	26 (23, 34)	0.748
ALT (U/L)	21 (16, 32)	25 (19, 30)	24 (20, 28)	22 (18, 30)	0.813
ALP (U/L)	64 (60, 70)	70 (63, 84)	77 (67, 88)	70 (63, 86)	0.399
Total Protein (g/dL)	6.9 (6.7, 7.3)	7.2 (6.8, 7.2)	7.1 (6.6, 7.5)	7.1 (6.6, 7.3)	0.834
Total Albumin (g/dL)	4.0 (3.8, 4.2)	4.2 (3.9, 4.4)	4.2 (3.9, 4.5)	4.1 (3.9, 4.4)	0.743
Glucose (mg/dL)	103 (100, 110)	106 (101, 114)	118 (102, 124)	108 (102, 120)	0.490
Total Cholesterol (mg/dL)	145 (119, 174)	190 (152, 212)	179 (144, 205)	176 (143, 206)	0.261
Triglyceride (mg/dL)	112 (96, 136)	104 (88, 156)	143 (99, 294)	112 (88, 206)	0.756
HDL (mg/dL)	41 (38, 46)	44 (38, 48)	42 (32, 53)	42 (35, 49)	0.810
LDL (mg/dL)	77 (63, 105)	118 (92, 129)	106 (70, 123)	102 (74, 126)	0.524
HbA1c (%)	5.7 (5.3, 6.4)	5.5 (5.3, 5.6)	5.9 (5.6, 6.6)	5.7 (5.4, 6.1)	0.251
C-Reactive Protein (mg/dL)	0.075 (0.046, 0.099)	0.119 (0.074, 0.181)	0.070 (0.058, 0.159)	0.081 (0.057, 0.162)	0.536
Magnesium (mEq/L)	2.10 (1.98, 2.23)	2.12 (2.01, 2.16)	2.09 (1.97, 2.17)	2.09 (1.97, 2.18)	0.857
Laboratory changes after drug administration					
WBC (×10^3^/μL)	1 (14.3%)	0 (0.0%)	0 (0.0%)	1 (4.2%)	0.282
Hemoglobin (g/dL)	0 (0.0%)	0 (0.0%)	2 (22.2%)	2 (8.3%)	0.162
Platelet (×10^3^/μL)	0 (0.0%)	0 (0.0%)	1 (11.1%)	1 (4.2%)	0.419
Blood Urea Nitrogen (mg/dL)	2 (28.6%)	2 (25.0%)	1 (11.1%)	5 (20.8%)	0.652
Creatinine (mg/dL)	0 (0.0%)	0 (0.0%)	2 (22.2%)	2 (8.3%)	0.162
Total Bilirubin (mg/dL)	0 (0.0%)	1 (12.5%)	1 (11.1%)	2 (8.3%)	0.635
AST (U/L)	0 (0.0%)	2 (25.0%)	1 (11.1%)	3 (12.5%)	0.34
ALT (U/L)	0 (0.0%)	0 (0.0%)	1 (11.1%)	1 (4.2%)	0.419
ALP (U/L)	0 (0.0%)	0 (0.0%)	0 (0.0%)	0 (0.0%)	_
Total Protein (g/dL)	0 (0.0%)	0 (0.0%)	0 (0.0%)	0 (0.0%)	_
Total Albumin (g/dL)	0 (0.0%)	0 (0.0%)	0 (0.0%)	0 (0.0%)	_
Glucose (mg/dL)	2 (28.6%)	2 (25.0%)	0 (0.0%)	4 (16.7%)	0.233
Total Cholesterol (mg/dL)	0(0.0%)	0 (0.0%)	0 (0.0%)	0 (0.0%)	_
Triglyceride (mg/dL)	0 (0.0%)	1 (12.5%)	1 (11.1%)	2 (8.3%)	0.635
HDL (mg/dL)	1 (14.3%)	2 (25.0%)	1 (11.1%)	4 (16.7%)	0.419
LDL (mg/dL)	0 (0.0%)	0 (0.0%)	0 (0.0%)	0 (0.0%)	_
HbA1c (%)	1 (14.3%)	2 (25.0%)	2 (22.2%)	5 (20.8%)	0.871
C-Reactive Protein (mg/dL)	0 (0.0%)	0 (0.0%)	0 (0.0%)	0 (0.0%)	_
Magnesium (mEq/L)	2 (28.6%)	2 (25.0%)	0 (0.0%)	4 (16.7%)	0.233

Continuous variables are presented as median (Q1, Q3); categorical variables are presented as n (%). Continuous variables were compared among the three groups using the Kruskal–Wallis test. Q1, first quartile; Q3, third quartile; SBP, Systolic Blood Pressure; DBP, Diastolic Blood Pressure; HR, Heart Rate; RR, Respiratory Rate; BT, Body temperature; HTN, Hypertension; DM, Diabetes Mellitus; CVD, Cerebrovascular disease; CLD, Chronic Liver Disease; CKD, Chronic Kidney Disease; CRD, Chronic Respiratory Disease; WBC, White Blood Cell; AST, Aspartate Aminotransferase; ALT, Alanine Aminotransferase; ALP, Alkaline Phosphatase; HDL, High-Density Lipoprotein; LDL, Low-Density Lipoprotein.

For microbiome analyses, differences in the Observed ASV richness and the Shannon index between baseline and week 24 were evaluated using the paired Wilcoxon signed-rank test. Beta diversity was assessed using Bray–Curtis dissimilarity calculated from the ASV-level abundance profiles. Differences in the microbial community composition between baseline and week 24 were evaluated separately within each treatment group using permutational multivariate analysis of variance (PERMANOVA) implemented with the adonis2 function in the vegan R package (Oksanen et al., 2026). Permutations were restricted to patient ID to account for the paired study design. In addition to the descriptive within-group PERMANOVA analyses, a formal PERMANOVA model including treatment group, visit, and their interaction was fitted using marginal tests in adonis2. The treatment group-by-visit interaction was used as the formal cross-arm inferential term; 9,999 permutations were restricted within patient ID to preserve the paired structure (**Supplementary Table S1**). The genus-level differential abundance between baseline and week 24 was assessed using Analysis of Compositions of Microbiomes with Bias Correction 2 (ANCOM-BC2) (Lin and Peddada, 2024). Each treatment group-visit combination was included as a fixed effect, and a patient-specific random intercept was included to account for repeated measurements within individuals. Before model fitting, taxa were filtered using a prevalence threshold of >5% and required to have nonzero counts in at least two patients. Taxa with zero variance were removed, and structural zeros were handled using ANCOM-BC2. To evaluate whether longitudinal taxonomic changes differed among the treatment groups while accounting for the baseline imbalances, the formal ANCOM-BC2 interaction model included treatment group, visit, their interaction, standardized baseline age, and standardized baseline blood urea nitrogen (BUN) as fixed effects and a patient-specific random intercept, (1|patient). Baseline BUN was defined as the V2 value and held constant across both observations from each participant; V4 BUN was not included because it was measured after treatment initiation. Multiple testing was controlled using the Benjamini–Hochberg method (p_adj_method = ‘BH’). Pairwise interaction coefficients quantified differences in V4-minus-V2 changes for P-CAB versus PPI, P-CAB versus the no acid-suppressive therapy group, and PPI versus the no acid-suppressive therapy group (**Supplementary Table S2**). Baseline microbiome comparability was additionally assessed using pretreatment V2 samples. Observed ASV richness and Shannon diversity were compared using the Kruskal–Wallis test. Baseline community composition was evaluated using ASV-level Bray–Curtis PERMANOVA with 9,999 unrestricted permutations, and homogeneity of multivariate dispersion was evaluated using PERMDISP. Genus-level baseline differential abundance was assessed using ANCOM-BC2 with treatment group as the fixed effect and Benjamini-Hochberg correction. Because each participant contributed only one baseline sample, neither within-subject permutation restriction nor a patient-specific random effect was applied to the baseline analyses (**Supplementary Figure S1**). Given the exploratory nature of the genus-level analysis, a nominal *p* < 0.10 was used as a reporting threshold for taxon-level signals. This threshold was not considered evidence of confirmatory statistical significance. Accordingly, taxonomic findings meeting this threshold were regarded as exploratory and hypothesis-generating rather than definitive differential-abundance findings.

## 3 Results

The demographic and clinical characteristics of the patients are summarized in **Table 1**. A total of 38 participants were enrolled and randomly assigned to the P-CAB (n = 12), PPI (n = 13), and no acid-suppressive therapy (n = 13) groups. Baseline fecal samples were not obtained from five participants: two in the P-CAB group and three in the PPI group. Among the remaining 33 participants, nine discontinued participations before week 24: six declined repeat fecal sampling (two in the P-CAB group, one in the PPI group, and three in the no acid-suppressive therapy group), and three withdrew for personal reasons (one in each group). Consequently, 24 participants with paired baseline and week-24 fecal samples were included in the final analysis: seven in the P-CAB group, eight in the PPI group, and nine in the no acid-suppressive therapy group (**Figure 1A**). No participant discontinued because of a drug-related adverse event. Baseline characteristics of completers and non-completers were generally comparable, although nominal differences were observed in ALT and total cholesterol levels (**Supplementary Table S3**). Given the exploratory comparisons and small sample size, these findings do not establish a clear pattern of systematic attrition bias.

### 3.1 Baseline characteristics

A comparison of the baseline characteristics among the 24 study participants revealed an age imbalance between the groups. The median age was 72.0 (68.5, 78.0) years in the P-CAB group, 59.5 (59.0, 62.2) years in the PPI group, and 70.0 (66.0, 76.0) years in the no acid-suppressive therapy group (*p* = 0.004). Although the PPI group had a numerically lower male proportion (62.5%) than the P-CAB (85.7%) and the no acid-suppressive therapy (77.8%) groups, this difference was not statistically significant (*p* = 0.670). Height did not differ significantly among the three groups (*p* = 0.065). Body weight, alcohol consumption, smoking status, and prevalence of underlying diseases (including hypertension, diabetes mellitus, and dyslipidemia) also showed no statistically significant differences among the three groups.

### 3.2 Baseline laboratory parameters

Analysis of baseline laboratory findings revealed a significant difference in BUN levels among the groups. Median BUN levels were 15.8 (14.8, 20.4) mg/dL in the P-CAB group, 12.0 (10.4, 13.6) mg/dL in the PPI group, and 14.1 (13.2, 17.4) mg/dL in the no acid-suppressive therapy group (*p* = 0.012). Other parameters, including WBC count, Hb level, creatinine level, liver function indicators (AST, ALT, ALP, and albumin levels), lipid profiles, and HbA1c levels, showed no significant differences among the groups.

### 3.3 Concomitant cardiovascular medications

All participants received dual antiplatelet therapy with aspirin and clopidogrel, and none received ticagrelor or prasugrel. All participants were also treated with statins. Statin intensity did not differ significantly among the P-CAB, PPI, and no acid-suppressive therapy groups (p = 0.322). The distributions of beta-blockers (p = 0.502), angiotensin-converting enzyme inhibitors or angiotensin receptor blockers (p = 0.117), and calcium channel blockers (p = 0.866) also did not differ significantly among the groups (**Supplementary Table S4**).

### 3.4 Laboratory changes following drug administration

Comparison of the incidence of laboratory abnormalities following drug administration revealed no statistically significant differences between the groups across all parameters (all *p* > 0.05). Changes in AST and glucose levels were observed in 25.0% (n = 2) of the PPI group; however, these rates were not statistically significant when compared with the P-CAB or no acid-suppressive therapy groups. Changes in creatinine levels, a marker of renal function, were not observed in either the P-CAB or PPI groups and were reported in only 22.2% (n = 2) of the no acid-suppressive therapy group (*p* = 0.162). No drug-related adverse events or episodes of gastrointestinal bleeding were reported during the 24-week follow-up period. No participant received antibiotics, probiotics/prebiotics, H2-receptor antagonists, or additional acid-suppressive medications outside the assigned study medication during the 24-week follow-up.

### 3.5 Baseline microbiome comparability among treatment groups

At baseline, neither Observed ASV richness (Kruskal–Wallis H = 0.221, df = 2, *p* = 0.895; **Supplementary Figure S1A**) nor Shannon diversity (H = 1.632, df = 2, *p* = 0.442; **Supplementary Figure S1B**) differed significantly among the three treatment groups. Baseline Bray–Curtis community composition also did not differ significantly among the groups (PERMANOVA R^2^ = 0.081, pseudo-F = 0.930, *p* = 0.695), and multivariate dispersion was not significantly different (PERMDISP F = 1.710, *p* = 0.205; **Supplementary Figure S1C**). The mean relative abundances of the 20 most abundant genus-level taxa are presented in **Supplementary Figure S1D**, with crosses indicating taxa classified by ANCOM-BC2 as structural zeros in the corresponding group. In the baseline ANCOM-BC2 analysis, none of the 176 non-structural-zero taxa tested by the model had a BH-adjusted q-value < 0.05 (minimum q = 0.055). However, 69 of 245 filtered genus-level taxa were flagged as structural zeros in at least one group. Given the small group sizes and the low prevalence of many taxa, these group-specific absences were interpreted cautiously; therefore, complete baseline taxonomic comparability cannot be assumed.

### 3.6 Changes in gut microbiota alpha diversity

When all groups were combined, the Observed ASV richness was significantly higher at V4 than at V2 (*p* = 0.003; **Figure 2A**). In the group-stratified analysis, the P-CAB group showed a significant increase in Observed ASV richness from V2 to V4 (*p* = 0.016), whereas no significant changes were observed in the PPI or no acid-suppressive therapy groups (**Figure 2B**). The Shannon index showed no significant differences between V2 and V4 either in the overall cohort or within any of the three study groups (**Figures 2A, B**). These findings indicate that the observed alpha-diversity change was primarily related to microbial richness rather than to the combined richness and evenness of the microbial community.

**Figure 2:**
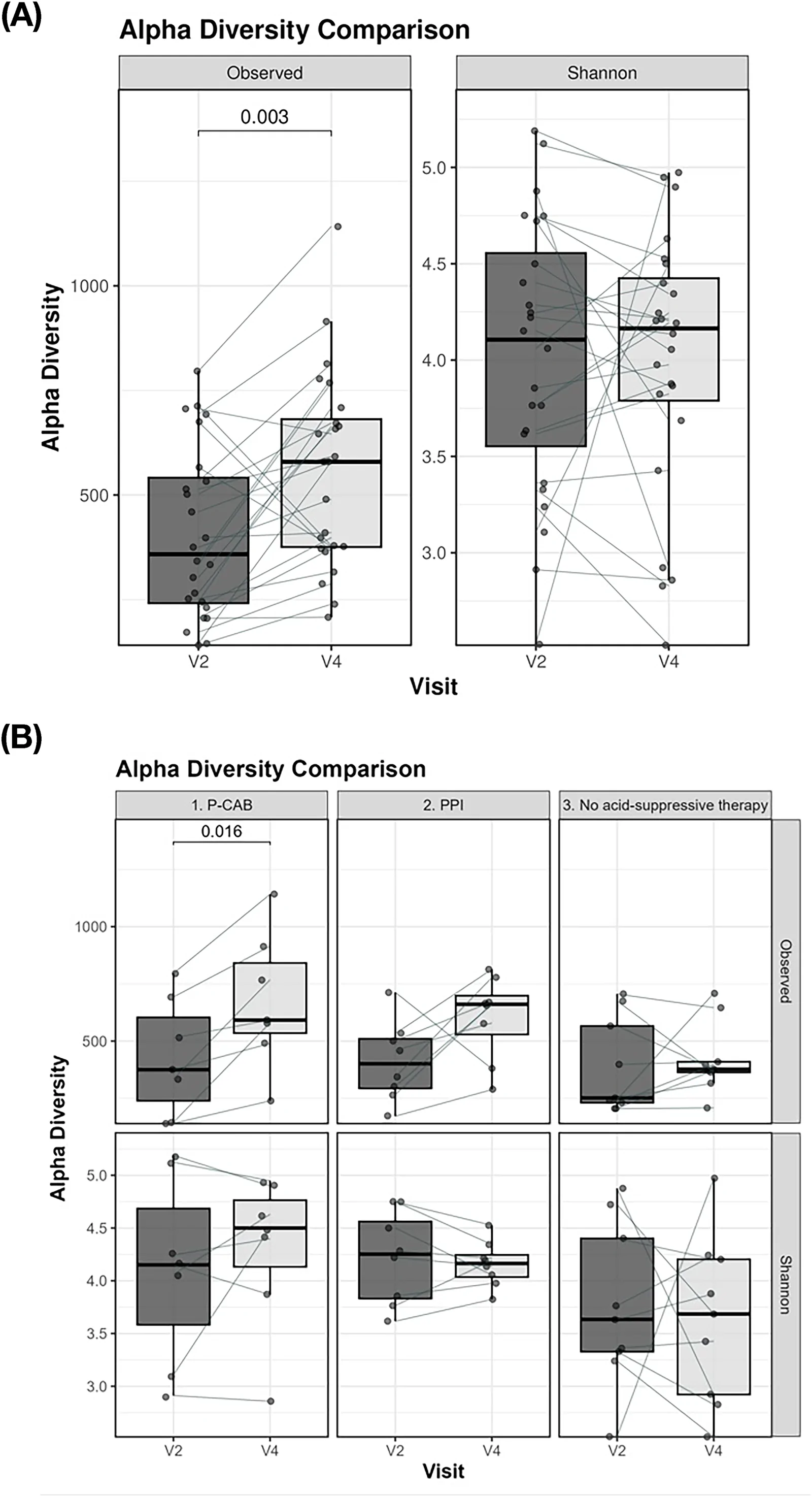
Comparison of alpha diversity between visits according to medication group. **(A)** Overall comparison of Observed and Shannon alpha diversity indices between V2 and V4. **(B)** Group-stratified comparison of Observed and Shannon indices between V2 and V4 in the P-CAB, PPI, and the no acid-suppressive therapy groups. Box plots show the median and interquartile range, with individual samples shown as points. Lines connect paired samples from the same patient. P-values were calculated using the paired Wilcoxon signed-rank test, and significant comparisons are annotated.

### 3.7 Changes in gut microbiota beta diversity

PCoA based on ASV-level Bray–Curtis dissimilarity showed substantial overlap between the V2 and V4 samples within each study group (**Figures 3A–C**). However, paired PERMANOVA with permutations restricted by patient ID revealed significant differences in microbial community composition between V2 and V4 in the P-CAB group (R² = 0.070, *p* = 0.031; **Figure 3A**) and PPI group (R² = 0.059, *p* = 0.008; **Figure 3B**). No significant differences were observed in the no acid-suppressive therapy group (R² = 0.021, *p* = 0.691; **Figure 3C**). The relatively small R² values indicate that the visit explained a modest proportion of the overall variation in microbial community composition. In the formal cross-arm model, the treatment group-by-visit interaction was not significant (R² = 0.021, pseudo-F = 0.505, p = 0.270), indicating that the beta-diversity trajectories did not differ detectably among the three groups (**Supplementary Table S1**).

**Figure 3:**
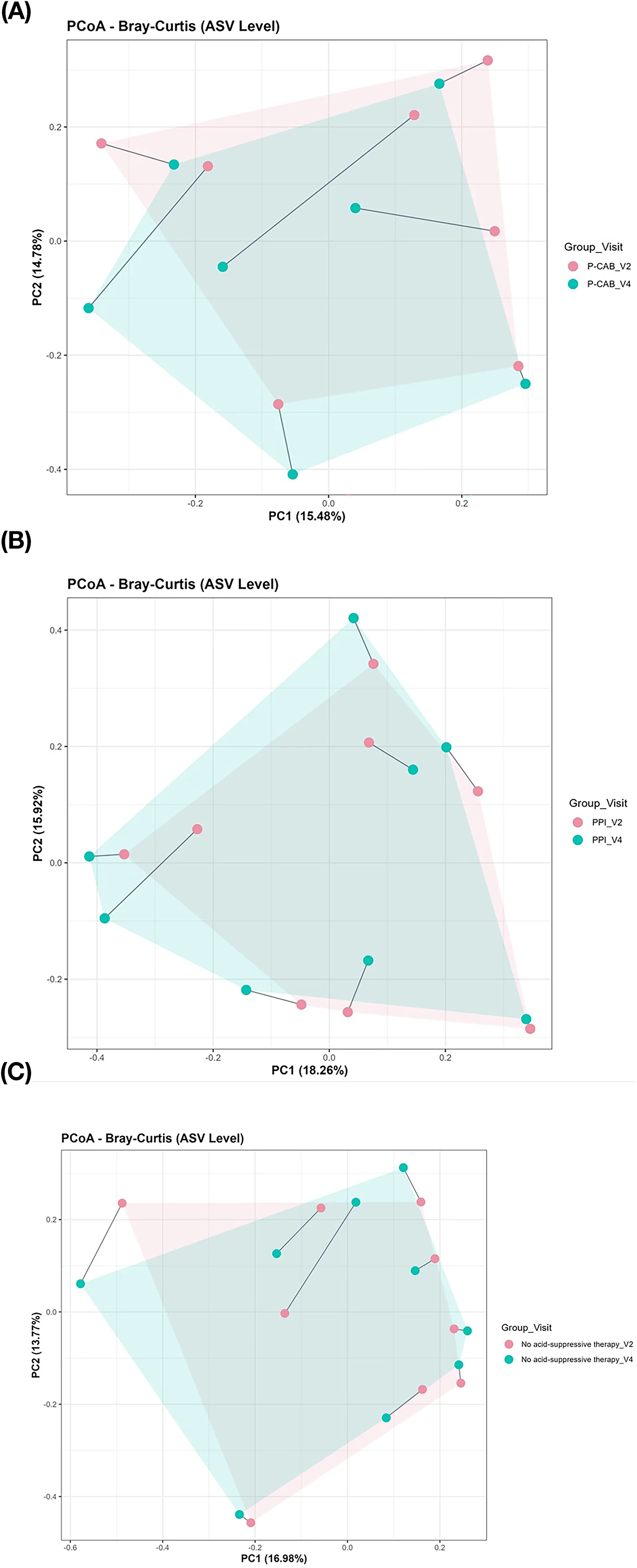
Principal coordinate analysis of beta diversity from V2 to V4 according to treatment group. Principal coordinate analysis (PCoA) plots based on ASV-level Bray–Curtis dissimilarities are shown for the **(A)** P-CAB, **(B)** PPI, and **(C)** the no acid-suppressive therapy groups. Points represent individual samples and are colored according to visit timepoint (V2 and V4). Lines connect the V2 and V4 samples from the same participant to illustrate individual paired trajectories. Shaded polygons represent convex hulls encompassing samples from each visit. Differences in microbial community composition between V2 and V4 were assessed using PERMANOVA with permutations restricted by patient ID to account for the paired study design. Significant differences were observed in the P-CAB group (R² = 0.070, *p* = 0.031) and the PPI group (R² = 0.059, *p* = 0.008), whereas no significant difference was observed in the no acid-suppressive therapy group (R² = 0.021, *p* = 0.691). A formal PERMANOVA model including treatment group, visit, and their interaction showed no significant treatment group-by-visit interaction (R² = 0.021, pseudo-F = 0.505, p = 0.270; 9,999 permutations restricted within patient ID; **Supplementary Table S1**).

### 3.8 Composition of gut microbiota

Stacked relative abundance plots at the family and genus levels showed substantial inter-individual variation in the microbial composition (**Figures 4A, B**). At the family level, Lachnospiraceae, Bacteroidaceae, Prevotellaceae, and Ruminococcaceae accounted for a substantial proportion of the microbial community (**Figure 4A**). At the genus level, the major taxa included *Bacteroides*, *Prevotella*, *Faecalibacterium*, *Blautia*, Escherichia-Shigella, and *Streptococcus* (**Figure 4B**). ANCOM-BC2 was used to compare genus-level abundance between V2 and V4 within each study group (**Figures 4C–E**). In the P-CAB group, *Streptococcus*, *Haemophilus*, *Negativibacillus*, and Lachnospiraceae UCG-004 were increased at V4, whereas the [*Eubacterium*] hallii group, *Anaerostipes*, the Lachnospiraceae ND3007 group, *Paraprevotella*, *Bifidobacterium*, *Megasphaera*, *Mitsuokella*, and *Clostridium* sensu stricto 1 were decreased (**Figure 4C**). In the PPI group, *Limosilactobacillus*, *Streptococcus*, *Veillonella*, *Haemophilus*, *Erysipelatoclostridium*, *Granulicatella*, and *Prevotella* were increased at V4, whereas the [*Ruminococcus*] gauvreauii and Lachnospiraceae ND3007 groups were decreased (**Figure 4D**). In the no acid-suppressive therapy group, *Hungatella*, *Intestinimonas*, *Streptococcus*, *Oscillibacter*, and *Eisenbergiella* were increased at V4, whereas *Dorea*, *Blautia*, Family XIII UCG-001, *Butyricicoccus*, the NK4A214 group, *Marvinbryantia*, *Terrisporobacter*, the [*Eubacterium*] hallii group, *Monoglobus*, the Lachnospiraceae ND3007 group, and the [*Ruminococcus*] gauvreauii group were decreased (**Figure 4E**). All taxon-level findings reported above met the exploratory reporting threshold of nominal p < 0.10. Given the small group sizes and the number of taxa examined, these findings should be regarded as hypothesis-generating rather than definitive evidence of differential abundance. In the formal age- and baseline-BUN-adjusted mixed-effects ANCOM-BC2 interaction analysis, 131 genus-level taxa were evaluated across three pairwise longitudinal contrasts. For taxa lacking a genus-level assignment, the lowest available higher taxonomic rank was used as the taxon label. No taxon had a BH-adjusted q-value < 0.05 or met the robust-significance criterion in any contrast (**Supplementary Table S2**). Thus, the nominal within-arm signals in **Figures 4C–E** do not establish differential treatment effects.

**Figure 4:**
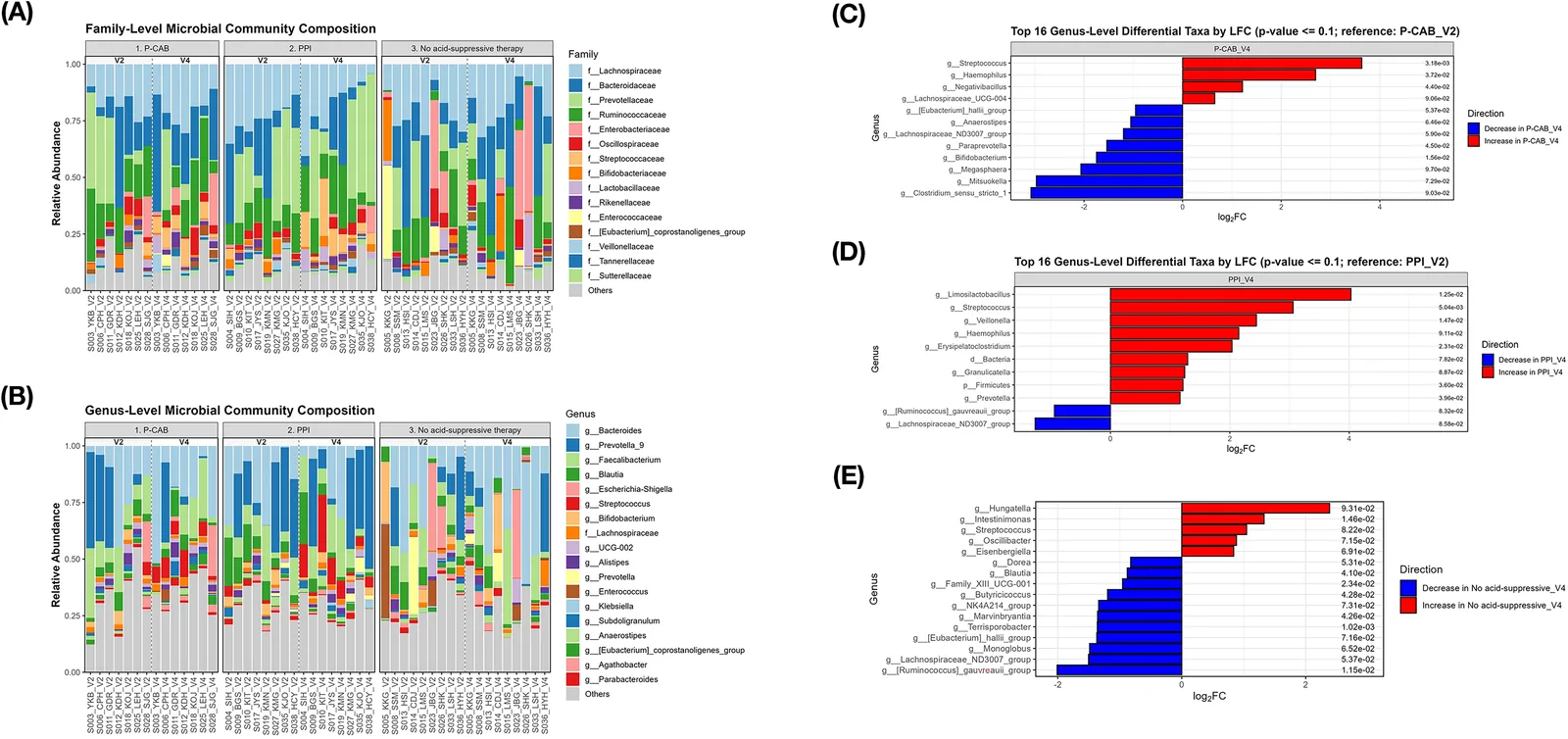
Taxonomic composition and differential abundance of the gut microbiome from V2 to V4 according to treatment group. **(A, B)** Stacked bar plots showing the relative abundance of microbial taxa at the family **(A)** and genus **(B)** levels in the P-CAB, PPI, and the no acid-suppressive therapy groups at V2 and V4. Each bar represents an individual sample, and colors indicate different taxa. Taxa with a mean relative abundance below 1% across the displayed dataset were combined into the “Others” category. Samples were ordered alphabetically within each study group and visit. For taxa without an assignment at the displayed taxonomic rank, the closest annotated higher taxonomic rank is shown. **(C–E)** Genus-level differential abundance analysis between V2 and V4 within the P-CAB **(C)**, PPI **(D)**, and no acid-suppressive therapy **(E)** groups, performed using ANCOM-BC2. V2 was used as the reference timepoint in each group. Taxa meeting the exploratory reporting threshold of nominal p < 0.10 are shown and ranked by log_2_ fold change. This threshold was used for exploratory visualization and does not denote confirmatory statistical significance. None of the reported taxa remained significant after Benjamini–Hochberg false discovery rate (FDR) correction. Red bars indicate taxa with higher estimated abundance at V4 than at V2, whereas blue bars indicate taxa with lower estimated abundance at V4. ANCOM-BC2 models treated each study group–visit combination as a fixed effect and included a patient-specific random intercept to account for repeated measurements within individuals. Before model fitting, taxa were filtered using a prevalence threshold of >5%, required nonzero counts in at least two patients, and taxa with zero variance were removed; structural zeros were handled within ANCOM-BC2.

## 4 Discussion

This prospective randomized controlled pilot study examined the longitudinal effects of two pharmacologically distinct acid-suppressive agents on the gut microbiota of patients with angina pectoris undergoing PCI who required DAPT. Although PPIs are routinely prescribed for gastrointestinal protection in this setting, mounting evidence indicates that sustained gastric acid suppression perturbs the composition and metabolic output of the resident gut microbiota. However, clinical data regarding the microbiome-modulating properties of P-CABs are scarce.

This study made three principal observations. Although statistically significant longitudinal changes were detected in microbial diversity, the overall shifts were modest in magnitude. The observed ASV richness increased significantly in the overall cohort and the P-CAB group, whereas the Shannon diversity index remained unchanged. In addition, paired PERMANOVA identified statistically significant within-subject shifts in microbial community composition in the P-CAB and PPI groups. However, the visit time point explained only 5.9–7.0% of the total community variation, indicating that these changes did not represent large-scale restructuring of the overall gut microbial community. This finding is consistent with several prospective interventional studies demonstrating that short- to medium-term acid-suppressive therapy does not substantially remodel the overall gut microbial richness or community-level structure. A 7-day course of esomeprazole in healthy adult volunteers did not alter the alpha or beta diversity of the gut microbiota, despite inducing significant taxon-level compositional changes, suggesting that the magnitude of microbiome variation attributable to acid-suppressive therapy may be relatively small compared to the inherent inter-individual differences (Kanno et al., 2009; Freedberg et al., 2015). In contrast, large-scale cross-sectional and cohort studies examining long-term PPI use have consistently reported significant compositional shifts in the gut microbiota. Imhann et al. demonstrated a significant reduction in Shannon diversity along with changes affecting 20% of bacterial taxa in a combined analysis of 1,815 individuals (Imhann et al., 2016; Zhang et al., 2023), Jackson et al. confirmed significant taxon-level alterations, including depletion of Lachnospiraceae and Ruminococcaceae in a large healthy twin cohort, even after adjusting for host genetics (Jackson et al., 2016), suggesting that diversity disruption is a time-dependent phenomenon requiring more prolonged or cumulative exposure. A meta-analysis of 16S rRNA gene sequencing datasets further confirmed a decrease in the Shannon diversity index in PPI users, along with the depletion of Ruminococcaceae and Lachnospiraceae, key short-chain fatty acid (SCFA)-producing families (Zhang et al., 2023). Clinical data on the effect of P-CABs on gut microbial diversity remain limited. A recent systematic review of vonoprazan-based regimens reported a significant reduction in alpha diversity. However, most of the included studies involved concomitant antibiotic therapy for *Helicobacter pylori* eradication, substantially confounding the extent to which the observed microbiota alterations could be attributed to the acid-suppressive agent itself rather than to antibiotic exposure (Ouyang et al., 2023). In the present study, the modest magnitude of the observed alpha- and beta-diversity changes most likely reflects the resilience of the gut microbial community to 24 weeks of standard-dose acid suppression. This pattern is consistent with prior interventional studies demonstrating that short-term PPI exposure preserves overall microbiome diversity despite inducing discrete taxonomic shifts (Freedberg et al., 2015; Bibbò et al., 2025). Notably, inter-individual microbiome variability substantially exceeded treatment-associated effects in both previous studies and the current pilot cohort. Taken together, the present findings suggest limited but detectable longitudinal changes rather than extensive disruption of the overall gut microbial community. It is important to distinguish statistical significance from biological or clinical importance in this setting. Although the paired PERMANOVA showed statistically significant within-group shifts in both the P-CAB and PPI groups, visit explained only 5.9–7.0% of the total variation in community composition, indicating that the longitudinal shifts were modest (Falony et al., 2016; Kelly et al., 2015). Similarly, the increase in observed ASV richness without a corresponding change in the Shannon index suggests that the alpha-diversity change reflected shifts in richness rather than a broader reorganization of community evenness (Shade, 2017). Importantly, an increase in richness should not necessarily be interpreted as beneficial, particularly because some of the taxonomic changes included enrichment of oral-associated organisms such as Streptococcus. Taken together, these findings are best interpreted as evidence of a detectable but limited compositional perturbation rather than a demonstrated biologically or clinically meaningful alteration of the gut microbiome. Consistent with this interpretation, the treatment group-by-visit interaction in the formal cross-arm PERMANOVA was not significant (R² = 0.021, p = 0.270). Therefore, the significant within-group tests in the P-CAB and PPI arms cannot be interpreted as evidence that their longitudinal trajectories differed from one another or from the no acid-suppressive therapy group.

Second, *Streptococcus* increased from baseline to week 24 in all three study groups. Although the estimated within-group increases were numerically larger in the P-CAB and PPI groups than in the no acid-suppressive therapy group, none of the age- and baseline-BUN-adjusted treatment group-by-visit contrasts remained significant after Benjamini–Hochberg correction. Therefore, the present findings do not establish that acid suppression caused the observed increase in *Streptococcus*. Nevertheless, the finding is biologically plausible because reduced gastric acidity may facilitate the passage of oral-associated microorganisms into the intestine. Previous studies of PPI users have reported enrichment of oral-associated taxa, including Streptococcus, and findings consistent with increased oral-to-gut microbial transmission (Imhann et al., 2016; Jackson et al., 2016; Xiao et al., 2024; Zhu et al., 2024). However, the present study did not collect oral samples or perform strain-level source tracking, and *Streptococcus* also increased in the no acid-suppressive therapy group. Accordingly, this finding should be regarded as a cohort-level longitudinal signal and a hypothesis for future studies using paired oral and fecal sampling, rather than as evidence of an acid-suppression effect or oral-to-gut microbial translocation.

Third, Lachnospiraceae-associated taxa and other potential SCFA-producing taxa showed mixed directions of change. Lachnospiraceae UCG-004 was increased in the P-CAB group, whereas the Lachnospiraceae ND3007 group, *Anaerostipes*, and the [*Eubacterium*] hallii group were decreased. The abundance of the Lachnospiraceae ND3007 group also decreased in the PPI and no acid-suppressive therapy groups. Lachnospiraceae is a butyrate-producing family within the phylum Firmicutes, which plays a central role in maintaining intestinal barrier integrity, modulating host immune responses, and conferring colonization resistance against enteric pathogens (Takiishi et al., 2017; Vacca et al., 2020). Collectively, the heterogeneous taxonomic changes observed within the P-CAB and PPI groups generate hypotheses regarding potentially distinct longitudinal patterns. However, because no treatment group-by-visit interaction remained significant after multiple-testing correction, these findings do not demonstrate differential microbiome effects between tegoprazan and pantoprazole. Previous meta-analyses have linked prolonged PPI use to Lachnospiraceae depletion and identified this as a potential marker of CDI susceptibility (Antharam et al., 2013; Macke et al., 2020). In our cohort, however, Lachnospiraceae-affiliated taxa changed in heterogeneous and, in some cases, opposite directions within the same arm, butyrate-producing capacity is not uniformly distributed across taxa within this family (Vital et al., 2017), and neither fecal SCFA concentrations, intestinal permeability, nor CDI incidence were measured. Accordingly, these taxonomic findings should be regarded as hypothesis-generating rather than as evidence of altered butyrate-producing capacity, barrier function, or CDI risk. The clinical relevance of microbiota alterations in this cohort may extend beyond gastrointestinal safety. Gut microbial metabolism generates multiple bioactive metabolites with potential cardiovascular effects. Trimethylamine N-oxide (TMAO), a gut microbiota–host co-metabolite derived from dietary choline and L-carnitine, has been linked to atherosclerosis, adverse cardiovascular events, platelet hyperreactivity, and thrombotic risk (Wang et al., 2011; Koeth et al., 2013; Tang et al., 2013; Zhu et al., 2016). Phenylacetylglutamine, another microbial–host co-metabolite generated from phenylalanine metabolism, has been associated with incident major adverse cardiovascular events and has been shown experimentally to enhance platelet responsiveness and thrombotic potential through adrenergic receptor signaling (Nemet et al., 2020). This pathway may be of particular interest in patients receiving dual antiplatelet therapy, in whom inhibition of platelet-mediated thrombosis is central to the prevention of ischemic events after PCI. Short-chain fatty acids may support epithelial barrier integrity and modulate inflammatory and metabolic responses, while experimental studies have implicated them in blood-pressure regulation and cardiac remodeling (Koh et al., 2016; Marques et al., 2017). Microbiota-modified bile acids can also influence host lipid, glucose, and inflammatory metabolism through bile acid receptors, including FXR and TGR5, although their effects depend on the specific bile acid species and host context (Wahlström et al., 2016). In a metagenome-wide association study, patients with atherosclerotic cardiovascular disease showed microbial compositional and functional signatures that differed from those of healthy controls (Jie et al., 2017), and the broader relationship between the gut microbiota and cardiovascular pathophysiology has been summarized in comprehensive reviews (Witkowski et al., 2020). Against this background, sustained, pharmacologically induced shifts in gut microbial composition after PCI may be relevant to cardiovascular as well as gastrointestinal outcomes. However, because microbial metabolites were not measured in the present study, these mechanistic links provide a rationale for further investigation rather than evidence derived from our current findings. Further studies with larger cohorts, direct functional measurements, and adequately powered treatment-by-visit interaction analyses are required to determine whether P-CABs and PPIs exert different effects on gut microbiome composition and function.

The pharmacological mechanisms of P-CABs and PPIs differ markedly, and may theoretically influence their mucosal protective profiles. PPIs require acid-dependent activation within the parietal cell canaliculi and exert their effect through irreversible inhibition of the H^+^/K^+^-ATPase pump. In contrast, P-CABs such as tegoprazan directly and reversibly inhibit the proton pump by competing with potassium ions, resulting in rapid and sustained acid suppression independent of meal timing and intragastric pH (Inatomi et al., 2016; Oshima and Miwa, 2018). These mechanistic distinctions have prompted interest in determining whether the two drug classes differ in their gastrointestinal safety profiles, including the risk of bleeding. In the present study, no upper or lower gastrointestinal bleeding events occurred in any of the three groups throughout the 24-week follow-up period. Given that patients receiving concomitant antiplatelet therapy represent a population with an inherently elevated risk of gastrointestinal bleeding, the absence of bleeding events, although observed across all three groups, including the no acid-suppressive therapy group, is consistent with a low short-term bleeding risk in this cohort, but does not permit conclusions regarding the comparative gastroprotective efficacy of the two acid-suppressive regimens. Nevertheless, the small sample size of the present pilot study precludes definitive comparisons, and larger, adequately powered trials are required to clarify whether the pharmacodynamic advantages of P-CABs translate into clinically meaningful differences in long-term gastrointestinal safety compared with conventional PPIs, particularly with regard to their differential effects on the gut microbiome.

This study has several limitations that should be considered when interpreting the findings. First, the limited sample size was further reduced by substantial attrition. Of the 38 randomized participants, five did not provide baseline fecal samples and nine discontinued participations before week 24, leaving 24 participants with paired fecal samples for the final analysis (7 in the P-CAB group, 8 in the PPI group, and 9 in the no acid-suppressive therapy group). Comparisons between completers and non-completers showed generally similar baseline characteristics, although nominal differences were observed in ALT and total cholesterol levels (**Supplementary Table S3**). Because these comparisons were exploratory and based on a small sample, systematic attrition bias cannot be excluded. The resulting small group sizes substantially limited the statistical power to detect modest differences in alpha and beta diversity metrics and fine-grained taxonomic shifts. Given the well-documented inter-individual variability of the human gut microbiome and the statistical constraints of a pilot-scale cohort, all findings, including the observed diversity patterns, the exploratory increase in Streptococcus in all three groups, and the taxonomic changes observed within the tegoprazan and pantoprazole groups, should be regarded as hypothesis-generating and interpreted with appropriate caution. Adequately powered multicenter randomized controlled trials are warranted to confirm and extend these preliminary observations. Second, significant baseline demographic and clinical imbalances were observed between the study groups, particularly with respect to age and BUN levels. Participants in the pantoprazole group were younger than those in the tegoprazan and the no acid-suppressive therapy group. This imbalance likely reflects chance variation associated with block randomization in a small pilot cohort. Because age is a well-established host factor associated with gut microbiota composition and diversity (Falony et al., 2016), the age imbalance represents an important potential confounder. Although the ANCOM-BC2 interaction model was adjusted for baseline age and BUN, the very small group sizes limited model precision and the ability to account for additional potential confounders. Residual confounding therefore cannot be excluded. Although the distributions of major concomitant cardiovascular medications did not differ significantly among the study groups, the small sample size limited our ability to exclude subtle medication-related confounding (**Supplementary Table S4**). Future adequately powered trials should consider stratified randomization by age or other major determinants of gut microbiome composition. Third, microbiota profiling was performed using 16S rRNA amplicon sequencing of the V3–V4 hypervariable region, a methodology that provides limited species-level resolution and does not permit direct functional characterization of the microbial community. Consequently, functionally important alterations, including changes in SCFA biosynthetic capacity, tryptophan and bile acid metabolism, and trimethylamine N-oxide (TMAO) pathways, could not be directly assessed. Future studies incorporating shotgun metagenomic sequencing and untargeted fecal metabolomics are necessary to validate the compositional differences reported here. Fourth, dietary intake and broader lifestyle factors were not systematically captured or controlled for throughout the study period. As dietary patterns are among the strongest known determinants of gut microbiota composition, the absence of a structured dietary assessment, including a validated food frequency questionnaire, represents a methodological limitation that may have introduced residual confounding factors. This concern is particularly relevant in the Korean context, given that consumption of fermented foods, including kimchi and doenjang, has been shown to significantly influence the relative abundance of Lachnospiraceae-affiliated genera and other SCFA-producing commensals. Notwithstanding these limitations, this prospective randomized pilot study provides an initial clinical characterization of longitudinal microbiome changes during P-CAB and conventional PPI treatment in a real-world cardiovascular population and establishes a preliminary evidence base upon which future large-scale multicenter trials with adequate power, strict dietary control, and functional microbiome profiling can be designed and conducted.

## 5 Conclusions

In this prospective, randomized pilot study of patients with angina pectoris undergoing PCI, we compared the longitudinal effects of tegoprazan and pantoprazole on the gut microbiota over 24 weeks. Overall community diversity remained largely stable, with a modest increase in richness and no change in the Shannon index. Exploratory increases in Streptococcus were observed over time in all three arms, with larger estimated changes in the P-CAB and PPI groups than in the no acid-suppressive therapy group; this pattern is consistent with, but does not demonstrate, oral-to-gut microbial translocation. Lachnospiraceae-associated taxa and other taxa previously linked to SCFA-related functions showed heterogeneous and, in some cases, opposing directions of change, with no consistent pattern of preservation or depletion in either treatment group. Neither the Bray–Curtis PERMANOVA interaction (R² = 0.021, p = 0.270) nor any genus-level ANCOM-BC2 interaction remained significant after BH correction, and no functional microbiome measurements were performed. Accordingly, these findings do not establish differential effects of tegoprazan and pantoprazole on the gut microbiota. Rather, they identify preliminary longitudinal microbiome signals that require confirmation in larger, adequately powered studies incorporating age-stratified randomization, improved control of potential confounders, and functional microbiome profiling using metagenomic and metabolomic approaches.

## Data Availability

The raw 16S rRNA gene amplicon sequencing data generated in this study have been deposited in the NCBI Sequence Read Archive (SRA) under BioProject accession PRJNA1517734 (SRA study accession SRP730272). The 48 SRA run accessions range from SRR40320586 to SRR40320633, and the corresponding BioSample accessions range from SAMN62667406 to SAMN62667453.

## Supplementary material

Supplementary Figure 1 Baseline microbiome comparisons among the treatment groups. Baseline (V2) samples collected before treatment initiation are shown for the P-CAB (n = 7), PPI (n = 8), and no acid-suppressive therapy (n = 9) groups. (A) Observed ASV richness and (B) Shannon diversity. (C) Principal coordinate analysis based on ASV-level Bray–Curtis dissimilarity. (D) Mean relative abundance of the 20 most abundant genus-level taxa. Crosses indicate taxa classified by ANCOM-BC2 as structural zeros in the corresponding group. Alpha diversity was compared using the Kruskal–Wallis test, community composition using PERMANOVA with 9,999 unrestricted permutations, and multivariate dispersion using PERMDISP.

ASV-level Bray–Curtis PERMANOVA including treatment group, visit, and their interaction. Bray–Curtis dissimilarities were calculated using ASV-level abundance data. A PERMANOVA model including treatment group, visit, and the treatment group-by-visit interaction was fitted with marginal (type III) tests for each term. The treatment group-by-visit interaction tests whether the change in microbial community composition from baseline (V2) to week 24 (V4) differed among the three study groups. A total of 9,999 permutations were performed and restricted within patient ID to preserve the paired structure of the data. The treatment group-by-visit interaction was not statistically significant. ASV, amplicon sequence variant; PERMANOVA, permutational multivariate analysis of variance; Df, degrees of freedom; SumOfSqs, sum of squares; R², proportion of variance explained; pseudo-F, PERMANOVA test statistic.

Supplementary Table 2 Age- and baseline BUN-adjusted ANCOM-BC2 analysis of treatment group-by-visit interactions among genus-level taxa. Genus-level differential-abundance analysis was performed using ANCOM-BC2. Treatment group, visit, their interaction, standardized baseline age, and standardized baseline blood urea nitrogen (BUN) were included as fixed effects, and a patient-specific random intercept, (1|patient), was included to account for repeated measurements. Baseline BUN was defined using the V2 measurement and was held constant across both observations from each participant. Taxa were retained if they had a prevalence >5%, nonzero counts in at least two participants, nonzero variance, and an estimable full adjusted mixed-effects model. Structural zeros were handled within ANCOM-BC2. Three pairwise interaction contrasts are reported for each of 131 retained taxa: P-CAB versus PPI, P-CAB versus no acid-suppressive therapy, and PPI versus no acid-suppressive therapy. Each interaction estimate represents the between-group difference in the change from baseline (V2) to week 24 (V4). Positive estimates indicate a greater longitudinal increase, or a smaller longitudinal decrease, in the first group named in the contrast; negative estimates indicate the opposite. Interaction estimates and standard errors are reported on both the natural-log and log_2_ scales, with 95% confidence intervals reported on the log_2_ scale. Raw p-values were adjusted within each contrast using the Benjamini–Hochberg method. Passed_sensitivity indicates that the ANCOM-BC2 pseudo-count sensitivity criterion was satisfied, and Robust_significant indicates significance after both BH correction and sensitivity assessment. No taxon had a BH-adjusted q-value < 0.05 or met the robust-significance criterion in any contrast. Taxa lacking an assigned genus-level annotation are reported using the closest available higher taxonomic rank. ANCOM-BC2, Analysis of Compositions of Microbiomes with Bias Correction 2; BUN, blood urea nitrogen; P-CAB, potassium-competitive acid blocker; PPI, proton pump inhibitor; ln, natural logarithm; log_2_, base-2 logarithm; SE, standard error; CI, confidence interval; W, Wald test statistic; BH, Benjamini–Hochberg; q-value, false discovery rate-adjusted p-value.

Supplementary Table 3 Baseline characteristics of completers and non-completers. Completers were randomized participants with paired baseline (V2) and week-24 (V4) fecal samples who were included in the final paired-sample analysis. Non-completers were randomized participants who were not included in the final analysis because a baseline fecal sample was not obtained or because they discontinued participation during the 24-week follow-up. Continuous variables are presented as mean ± standard deviation and were compared using the Mann–Whitney U test. Categorical variables are presented as n (%) or n/N (%) and were compared using Fisher’s exact test; randomized-arm distributions were compared using the Fisher–Freeman–Halton exact test. One completer had missing baseline comorbidity data; therefore, percentages for underlying diseases in the completer group were calculated using the available denominator of 23. ALT and total cholesterol showed nominal between-group differences. Because these comparisons were exploratory, involved multiple baseline variables, and were based on a small sample, the findings should be interpreted cautiously and do not exclude more subtle systematic attrition bias. A two-sided p-value < 0.05 was considered nominally significant. P-CAB, potassium-competitive acid blocker; PPI, proton pump inhibitor; SD, standard deviation; BMI, body mass index; SBP, systolic blood pressure; DBP, diastolic blood pressure; HR, heart rate; WBC, white blood cell count; BUN, blood urea nitrogen; AST, aspartate aminotransferase; ALT, alanine aminotransferase; HbA1c, glycated hemoglobin; CRP, C-reactive protein.

Supplementary Table 4 Distribution of concomitant cardiovascular medications according to treatment group. Values are presented as n (%). All participants received dual antiplatelet therapy with aspirin and clopidogrel, and none received ticagrelor or prasugrel. All participants also received statin therapy, although statin intensity varied among participants. Categorical variables were compared among the three study groups using the Fisher–Freeman–Halton exact test. For statin intensity, the p-value represents the overall comparison of the distributions of high-, moderate-, and low-intensity statin therapy. P-values were not calculated for medications with no between-group variation. P-CAB, potassium-competitive acid blocker; PPI, proton pump inhibitor; ACEi, angiotensin-converting enzyme inhibitor; ARB, angiotensin receptor blocker; CCB, calcium channel blocker.

Supplementary Table 5 Sample-level read-processing statistics. Imported merged reads represent PEAR-assembled reads imported into QIIME 2. Reads retained after initial q-score filtering were subsequently used as input for DADA2 denoising. Non-chimeric reads represent the final reads retained after DADA2 filtering, denoising, and chimera removal.
