# Supplementary material for: Effect of a potassium-competitive acid blocker on the gut microbiota in patients undergoing percutaneous coronary intervention: a randomized pilot study
Source: Front Cell Infect Microbiol. 2026 Oct 12;16:1942212. doi: 10.3389/fcimb.2026.1942212 (PMC13696315; doi:10.3389/fcimb.2026.1942212)
Supplement: Supplementary file 1 [file Table3.docx]

**Supplementary Table S3. Baseline comparison of completers versus non-completers.**

| **Variable** | **Completers (n = 24)** | **Non-completers (n = 14)** | **p-value** |
| --- | --- | --- | --- |
| **Demographics** | | | |
| Randomized arm, n | P-CAB: 7; PPI: 8; no acid-suppressive therapy: 9 | P-CAB: 5; PPI: 5; no acid-suppressive therapy: 4 | 0.915 |
| Male sex, n (%) | 18 (75.0%) | 8 (57.1%) | 0.296 |
| Age, mean ± SD (years) | 68.17 ± 9.13 | 69.57 ± 11.27 | 0.916 |
| Height (cm) | 162.02 ± 10.00 | 161.68 ± 11.35 | 0.797 |
| Weight (kg) | 65.38 ± 10.47 | 68.26 ± 16.42 | 0.449 |
| BMI (kg/m²) | 25.03 ± 4.26 | 25.79 ± 3.83 | 0.269 |
| Alcohol consumption, n (%) | 10 (41.7%) | 5 (35.7%) | 1.000 |
| Smoking status, n (%) | 6 (25.0%) | 2 (14.3%) | 0.684 |
| **Vital Signs** | | | |
| SBP (mmHg) | 130.54 ± 10.10 | 129.14 ± 14.94 | 0.976 |
| DBP (mmHg) | 80.17 ± 8.25 | 81.57 ± 10.64 | 0.523 |
| HR (beats/min) | 72.25 ± 8.92 | 77.71 ± 11.01 | 0.081 |
| **Underlying Disease, n/N (%)** | | | |
| Hypertension | 12/23 (52.2%) | 9/14 (64.3%) | 0.515 |
| Diabetes mellitus | 6/23 (26.1%) | 5/14 (35.7%) | 0.713 |
| Dyslipidemia | 7/23 (30.4%) | 7/14 (50.0%) | 0.303 |
| Cardiovascular disease | 1/23 (4.3%) | 0/14 (0.0%) | 1.000 |
| Chronic liver disease | 0/23 (0.0%) | 0/14 (0.0%) | 1.000 |
| Chronic kidney disease | 0/23 (0.0%) | 2/14 (14.3%) | 0.137 |
| **Baseline Laboratory Findings** | | | |
| WBC (×10³/μL) | 6.87 ± 2.01 | 6.41 ± 1.87 | 0.628 |
| Hemoglobin (g/dL) | 14.32 ± 1.31 | 13.15 ± 2.24 | 0.112 |
| Platelet (×10³/μL) | 198.75 ± 41.17 | 210.50 ± 63.95 | 0.296 |
| BUN (mg/dL) | 15.07 ± 3.87 | 15.01 ± 5.57 | 0.515 |
| Creatinine (mg/dL) | 0.85 ± 0.21 | 0.91 ± 0.27 | 0.545 |
| AST (U/L) | 31.75 ± 15.54 | 37.14 ± 13.13 | 0.096 |
| ALT (U/L) | 25.54 ± 10.48 | 36.57 ± 17.32 | **0.029** |
| Glucose (mg/dL) | 118.21 ± 34.96 | 125.21 ± 39.94 | 0.868 |
| Total cholesterol (mg/dL) | 174.33 ± 46.46 | 146.07 ± 34.11 | **0.025** |
| Triglyceride (mg/dL) | 146.88 ± 84.60 | 129.57 ± 68.97 | 0.716 |
| HbA1c (%) | 6.01 ± 1.04 | 6.51 ± 1.08 | 0.051 |
| C-reactive protein (mg/dL) | 0.16 ± 0.25 | 0.27 ± 0.40 | 0.458 |

Completers were randomized participants with paired baseline (V2) and week-24 (V4) fecal samples included in the primary analysis (P-CAB, n = 7; PPI, n = 8; no acid-suppressive therapy, n = 9). Non-completers were randomized participants who were not included in the final paired-sample analysis because baseline fecal samples were not obtained or they withdrew during the 24-week follow-up (P-CAB, n = 5; PPI, n = 5; no acid-suppressive therapy, n = 4). Continuous variables are presented as mean ± SD and were compared using the Mann–Whitney U test. Categorical variables are presented as n (%) or n/N (%) and were compared using Fisher’s exact test; the randomized-arm distribution was compared using the Fisher–Freeman–Halton exact test. One completer had missing baseline comorbidity data; percentages for underlying diseases therefore use the available denominator (N = 23). A two-sided p < 0.05 was considered nominally significant. ALT and total cholesterol showed nominal between-group differences; because these comparisons were exploratory and involved multiple baseline variables, they should be interpreted cautiously and do not by themselves establish systematic attrition bias. The small sample size also limits the ability to exclude more subtle baseline imbalances.
