# Supplementary material for: Effect of a potassium-competitive acid blocker on the gut microbiota in patients undergoing percutaneous coronary intervention: a randomized pilot study
Source: Front Cell Infect Microbiol. 2026 Oct 12;16:1942212. doi: 10.3389/fcimb.2026.1942212 (PMC13696315; doi:10.3389/fcimb.2026.1942212)
Supplement: Supplementary file 2 [file Table4.docx]

**Supplementary Table S4. Concomitant cardiovascular medications according to treatment group**

| **Medication** | **P-CAB (n = 7)** | **PPI (n = 8)** | **no acid-suppressive therapy group (n = 9)** | **p-value** |
| --- | --- | --- | --- | --- |
| **Aspirin** | 7 (100%) | 8 (100%) | 9 (100%) | — |
| **Clopidogrel** | 7 (100%) | 8 (100%) | 9 (100%) | — |
| Ticagrelor / Prasugrel | 0 (0%) | 0 (0%) | 0 (0%) | — |
| **Statin use** | 7 (100%) | 8 (100%) | 9 (100%) | — |
| **Statin intensity** |  |  |  | **0.322** |
| High intensity | 0 (0%) | 2 (25.0%) | 1 (11.1%) | — |
| Moderate intensity | 7 (100%) | 6 (75.0%) | 6 (66.7%) | — |
| Low intensity | 0 (0%) | 0 (0%) | 2 (22.2%) | — |
| **Beta-blocker** | 0 (0%) | 2 (25.0%) | 2 (22.2%) | 0.502 |
| **ACEi / ARB** | 5 (71.4%) | 2 (25.0%) | 2 (22.2%) | 0.117 |
| **Calcium channel blocker (CCB)** | 3 (42.9%) | 2 (25.0%) | 3 (33.3%) | 0.866 |

Values are presented as n (%). All participants received dual antiplatelet therapy with aspirin and clopidogrel. Categorical variables were compared among the three groups using the Fisher–Freeman–Halton exact test. For statin intensity, the p-value represents the overall comparison of the high-, moderate-, and low-intensity distributions. P-values were not calculated for variables with no between-group variation. P-CAB, potassium-competitive acid blocker; PPI, proton pump inhibitor; ACEi, angiotensin-converting enzyme inhibitor; ARB, angiotensin receptor blocker; CCB, calcium channel blocker.
