# Supplementary material for: Effect of a potassium-competitive acid blocker on the gut microbiota in patients undergoing percutaneous coronary intervention: a randomized pilot study
Source: Front Cell Infect Microbiol. 2026 Oct 12;16:1942212. doi: 10.3389/fcimb.2026.1942212 (PMC13696315; doi:10.3389/fcimb.2026.1942212)
Supplement: Supplementary file 3 [file Table5.docx]

**Supplementary Table S1.** **ASV-level Bray–Curtis PERMANOVA including treatment group, visit, and their interaction**

| **Term** | **Df** | **SumOfSqs** | **R^2^** | **Pseudo_F** | **P_value** |
| --- | --- | --- | --- | --- | --- |
| Group:Visit | 2 | 0.36308 | 0.021427 | 0.505024 | 0.2704 |
| Residual | 42 | 15.09768 | 0.890998 | NA | NA |
| Total | 47 | 16.94469 | 1 | NA | NA |
